# A patient with McLeod syndrome showing involvement of the central sensorimotor tracts for the legs

**DOI:** 10.1186/s12883-019-1526-9

**Published:** 2019-11-27

**Authors:** Takenobu Murakami, Dan Abe, Hideyuki Matsumoto, Ryo Tokimura, Mitsunari Abe, Amanda Tiksnadi, Shunsuke Kobayashi, Chikako Kaneko, Yuka Urata, Masayuki Nakamura, Akira Sano, Yoshikazu Ugawa

**Affiliations:** 1 Department of Neurology, Fukushima Medical University, Fukushima, Japan; 2 Department of Neurology, Tottori Prefectural Kousei Hospital, Kurayoshi, Japan; 3 Department of Neurology, Mitsui Memorial Hospital, Tokyo, Japan; 4 Center for Neurological Disorders, Fukushima Medical University, Fukushima, Japan; 5 Department of Neurology, Southern Tohoku General Hospital, Koriyama, Japan; 6 Department of Psychiatry, Kagoshima University Graduate School of Medical and Dental Sciences, Kagoshima, Japan; 7 Department of Neuro-regeneration, Fukushima Medical University, Fukushima, Japan

**Keywords:** McLeod syndrome, Transcranial magnetic stimulation, Central motor conduction time, Motor-evoked potential, Somatosensory-evoked potential

## Abstract

**Background:**

McLeod syndrome is a rare X-linked recessive acanthocytosis associated with neurological manifestations including progressive chorea, cognitive impairment, psychiatric disturbances, seizures, and sensorimotor axonal polyneuropathy. However, no studies have investigated the functioning of central sensorimotor tracts in patients with McLeod syndrome.

**Case presentation:**

A 66-year-old man had experienced slowly progressive chorea and gait disturbance due to lower limb muscle weakness since his early fifties. Blood examinations showed erythrocyte acanthocytosis and the reduction of Kell antigens in red blood cells. Brain magnetic resonance imaging showed atrophy of the bilateral caudate nuclei and putamen. The diagnosis of McLeod syndrome was confirmed by the presence of a mutation of the *XK* gene on the X chromosome. Somatosensory-evoked potential and transcranial magnetic stimulation studies demonstrated that the central sensory and motor conduction times were abnormally prolonged for the lower extremity but normal for the upper extremity.

**Conclusions:**

This is the first report of the involvement of the central sensorimotor tracts for the legs in a patient with McLeod syndrome. The clinical neurophysiological technique revealed the central sensorimotor tracts involvements clinically masked by neuropathy.

## Background

McLeod syndrome is an extremely rare progressive X-linked recessive type of neuroacanthocytosis that was first reported by Allen et al. in 1961 [1]. This syndrome is a multisystem disorder with central nervous system (CNS), neuromuscular, cardiovascular, and hematological manifestations. Red blood cell acanthocytosis in McLeod syndrome is associated with absent expression of the Kx antigen and reduced expression of the Kell antigen on the surface membranes of erythrocytes, which are caused by truncation or no expression of XK protein. The detection of mutations in the *XK* gene confirms a diagnosis of McLeod syndrome. The XK protein plays pivotal roles in organogenesis, cellular structure, and nutrient exchanges [2]. Patients with McLeod syndrome lack expression of this protein, which leads to acanthocytosis and neural degeneration.

Neurological symptoms in McLeod syndrome are various, including progressive chorea, cognitive impairment, psychiatric disturbances, and seizures [3]. Sensorimotor axonal neuropathy is also a typical clinical feature, which leads to distal-dominant muscular weakness with muscular atrophy. A previous pathological study using a mouse model of McLeod syndrome found axonopathy in the spinal cord and the sciatic nerve [4]. However, whether the central sensorimotor tracts are involved in McLeod syndrome remains unclear. For the present study we hypothesized that the central sensorimotor tracts are involved in this disorder.

We used two electrophysiological methods to evaluate the conduction of CNS pathways in McLeod syndrome. First, the central sensory conduction time (CSCT) was measured by recording median and tibial somatosensory-evoked potentials (SEPs). The latencies of the following components were identified: N9 (Erb’s point), N11, N13 (spinal dorsal horn), and N20 (primary sensory cortex) for the median SEP; and N8 (near-field potential of the tibial nerve at the popliteal fossa), N21 (L5–S1 dorsal horn), and P38 (primary sensory cortex) for the tibial SEP (see Table 2 for the montage). The CSCT is calculated as the latency difference between cortical and spinal components. Second, the central motor conduction time (CMCT) was measured using transcranial magnetic stimulation (TMS). TMS can noninvasively elicit motor-evoked potentials (MEPs) by stimulation of the motor cortex or spinal nerve roots; for example, TMS at neural foramina at the C7 and L5 levels elicits MEPs of hand muscles and leg muscles, respectively. The CMCT is defined as the latency difference of MEPs between motor cortical stimulation and spinal root stimulation [5]. Precisely speaking, the CMCT does not purely consist of the corticospinal component, instead including some peripheral component from the nerve root inside the spinal canal. The peripheral component is estimated to be around 0.6 ms for upper-limb muscles, and 1.5 ms or longer for lower-limb muscles since it includes the cauda equina. To overcome the unignorable cauda equina component, we recently reported a new CMCT parameter for the leg muscles named the cortico-conus motor conduction time (CCCT) [6], which is calculated as the MEP latency difference between cortical stimulation and conus stimulation (L1 level). The CCCT can estimate the true central motor conduction without including peripheral components. The new method also allows the cauda equina conduction time (CECT) to be measured, which is defined as the MEP latency difference between stimulation at the L5-level spinal root and L1-level conus [7, 8]. We applied this new TMS method to a patient with McLeod syndrome.

This is the first study to systematically examine the central conduction times in McLeod syndrome. We found significant prolongation of the central conduction for the leg muscles, suggesting that the syndrome involves not only peripheral nerves but also the central sensorimotor tracts.

## Case presentations

A 66-year-old man noticed involuntary movements in all extremities and weakness in the lower limb muscles in his early fifties. He had no particular family or past medical history. He was admitted to our hospital with a chief complaint of gait disturbance. On examination, he was conscious and fully oriented, but restless and irritable. He exhibited facial grimacing but no lip or tongue biting. He had chorea in all extremities. He also had right-side-dominant, distal-dominant muscular weakness with muscular atrophy (Medical Research Council Scale grade 1 for the tibialis anterior (TA) muscle and gastrocnemius muscle on the right side and 3 on the left side). His vibratory perception was impaired at the ankles, whereas superficial sensations were intact. He showed a positive Romberg’s sign. Deep tendon reflexes were absent in the extremities, and plantar reflex was indifferent. He needed a cane support in walking.

**Fig. 1:**
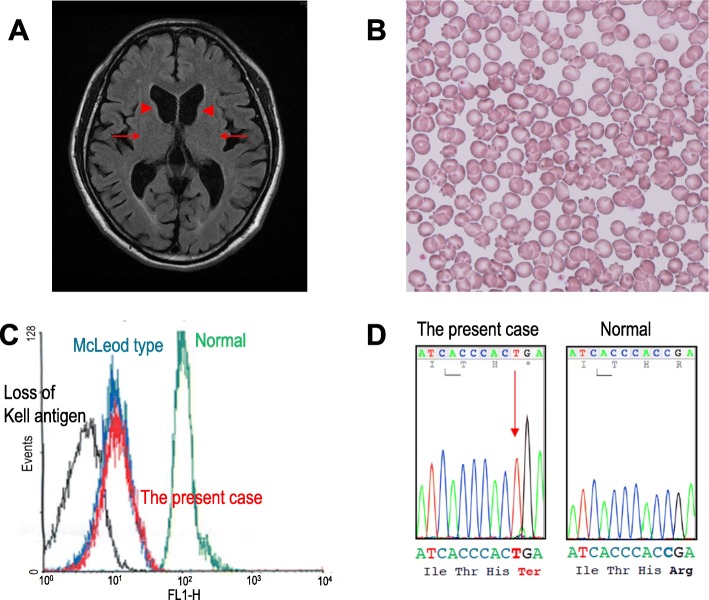
**a** MRI fluid-attenuated inversion recovery axial image shows atrophy of the caudate nuclei (arrowheads) and putamen (arrows) bilaterally. **b** Erythrocyte acanthocytosis present in a peripheral blood smear. **c** Flow cytometry revealed reduced Kell red blood cell antigens in the peripheral blood (red peak indicates the present case). **d** Sequencing of the *XK* gene disclosed mutation c.397C > T (p.Arg133Ter) in exon 2

Blood chemical examinations showed elevations of creatine phosphokinase (1609 U/l), aspartate transaminase (54 U/l), alanine transaminase (78 U/l), and lactate dehydrogenase (316 mg/dl). The electrocardiography and chest X-ray findings were normal. The brain magnetic resonance images (MRIs) revealed atrophy of bilateral caudate nuclei and putamen (Fig. 1a), but spinal MRIs showed no abnormalities. Single-photon-emission computed tomography with N-isopropyl-p-[123I]-iodoamphetamine revealed decreases in the blood flow in the basal ganglia.

Table 1 presents the results of the nerve conduction studies. The amplitudes of the compound motor action potentials were reduced in the tibial and fibular nerves, while the amplitudes of the sensory nerve action potentials were reduced in the sural nerves. Needle electromyographic examinations performed at the TA muscle and rectus femoris muscle revealed high-amplitude and long-duration motor unit potentials (MUPs). MUP recruitment was reduced during volitional contraction and fibrillation potentials were present at rest. These findings indicate the presence of chronic denervation and reinnervation processes.

**Table 1:** Results of nerve conduction studies

Motor nerve conduction study				
Nerve	Distal latency	Amplitude (mV)		Velocity
	(ms)	distal	proximal	(m/s)
Right Median	4.1	11.3	10.3	53
Right Ulnar	2.4	6.0	4.4	60
Right Tibial	5.1	***4.3*** (normal > 7.0)	***3.3*** (normal > 7.0)	44
Left Tibial	4.3	***6.5*** (normal > 7.0)	***3.9*** (normal > 7.0)	45
Right Fibular	5.0	***0.6*** (normal > 0.6)	***0.5*** (normal > 0.6)	36
Left Fibular	2.6	3.8	3.7	40
Sensory nerve conduction study				
	Distal latency	Amplitude (μV)		Velocity
	(ms)	distal	proximal	(m/s)
Right Median	3.1	11.2	3.1	60
Right Ulnar	3.3	5.2	0.8	54
Right Sural	2.8	***3.0*** (normal > 15.0)		50
Left Sural	3.4	***3.0*** (normal > 15.0)		42

The bold Italic values indicate under the normal limits

We examined the median and tibial SEPs (Table 2). The right median nerve SEPs showed no delay in any component and no prolongation of CSCT. In the right tibial nerve SEPs, the peripheral components had a normal latency, but the cortical latency was prolonged. CSCT was also abnormally prolonged (19.5 ms; normal < 13.2 ms).

**Table 2:** Results of SEP study

SEPs with median nerve stimulation				SEPs with tibial nerve stimulation			
Potential	Montage	Latency (ms)	Normal limit (ms)	Potential	Montage	Latency (ms)	Normal limit (ms)
(1) N9o	EPi-EPc	9.6	9.7	(1) N8o	Pfi-K	7.4	8.7
(2) N11o	C5s-Fz	11.1	11.6	(2) N21	L1 s-Icc	21.2	26.7
(3) N13o	C5s-Fz	13.3	13.7	(3) P38o	Cz’-Fz	***40.7***	38.2
(4) N20o	C3’-Fz	17.4	18.0	(4) P38	Cz’-Fz	***47.9***	44.7
		Conduction time (ms)				Conduction time (ms)	
CSCT [(4)–(3)]		4.1	4.8	CSCT [(3)–(2)]		***19.5***	13.2

*SEP* somatosensory-evoked potential, *CSCT* central sensory conduction time

*EPi* ipsilateral Erb’s point, *EPc* contralateral Erb’s point

*Pfi* ipsilateral popliteal fossa, *K* ipsilateral medial popliteal fossa, *Icc* contralateral iliac crest

The bold Italic values indicate over the normal limits

**Fig. 2:**
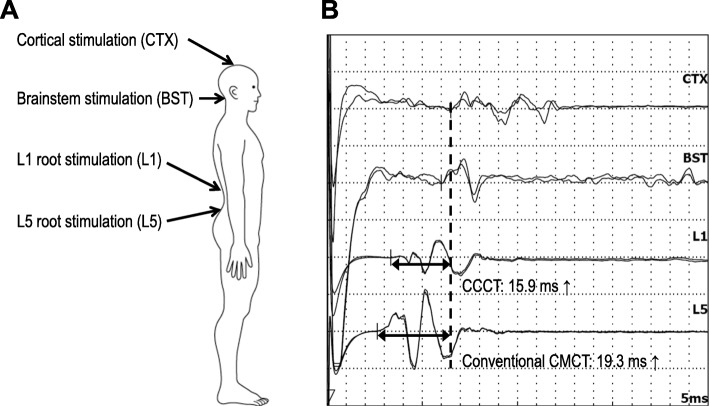
TMS findings. **a** Schematic of sites where magnetic stimulation was applied. **b** MEP latencies for cortical and brainstem stimulation are prolonged, while those for stimulation at levels L1 (conus) and L5 (neuro-foramina) are within the normal ranges. Both the conventional CMCT and the CCCT are abnormally prolonged

We used TMS to examine MEPs from the right first-dorsal interosseous (FDI) muscle and TA muscle (Table 3). MEP latencies for the FDI were normal for stimulation to the cortex, brainstem, and cervical nerve root. The CMCT was also within the normal range (6.5 ms; normal < 7.7 ms) [9]. MEP could not be evoked from the right TA muscle due to severe muscle atrophy, and so we recorded MEPs from the left TA muscle. MEP latencies were prolonged for stimulation to the cortex and brainstem, but normal for stimulation at levels L1 and L5. Both the conventional CMCT (19.3 ms; normal < 17.0 ms) and the CCCT (15.9 ms; normal < 14.7 ms) were abnormally prolonged [6] (Fig. 2). The CECT was normal.

**Table 3:** Results of TMS study

MEPs from FDI muscle			MEPs from TA muscle		
Stimulation site	Latency (ms)	Normal limit (ms)	Stimulation site	Latency (ms)	Normal limit (ms)
(1) Cortex	21.7	22.6	(1) Cortex	***32.6***	29.3
(2) Brainstem	18.8	18.8	(2) Brainstem	***30.1***	25.4
(3) Cervical root	15.2	15.2	(3) L1 root	16.7	16.8
	Conduction time (ms)		(4) L5 root	13.3	13.3
CMCT [(1)–(3)]	6.5	7.7		Conduction time (ms)	
			CMCT [(1)–(4)]	***19.3***	17.0
			CCCT [(1)–(3)]	***15.9***	14.7
			CECT [(3)–(4)]	3.4	4.4

*TMS* transcranial magnetic stimulation, *FDI* first-dorsal interroseous, *TA* tibialis anterior;

*CMCT* central motor conduction time, *CCCT* cortico-conus conduction time, *CECT* cauda equina conduction time

The bold Italic values indicate over the normal limits

The peripheral blood smear disclosed frequent acanthocytes (28% of red cells, Fig. 1b), and flow cytometry disclosed weak expression of Kell red blood cell antigens (Fig. 1c). On the basis of progressive chorea, psychiatric symptoms, sensorimotor axonal neuropathy in the lower extremities, and acanthocytosis, we performed gene sequencing of his blood samples. Sequence analysis of the *XK* gene detected mutation c.397C > T (p.Arg133Ter) in exon 2 (reference sequence NM_021083.2) (Fig. 1d). According to this gene mutation, he was diagnosed with McLeod syndrome [10].

He continued to take oral haloperidol at a dosage of 1.5 mg/day, and his chorea and restlessness were found to be well controlled in outpatient clinic examinations.

## Discussion and conclusions

Our patient had progressive chorea, psychiatric disturbances, distal-dominant muscular weakness with atrophy in the lower extremities, and acanthocytosis, which match the clinical features of McLeod syndrome. The diagnosis was confirmed by detection of a previously known mutation in the *XK* gene [3, 11, 12]. Nerve conduction studies showed sensorimotor axonal neuropathy only in the lower extremities. The cortical latency of the SEPs was also delayed for tibial nerve stimulation, suggesting the involvement of the central sensory pathway that includes the dorsal cord, medial lemniscus, thalamus, and primary sensory cortex. In addition, the TMS technique revealed the involvement of the corticospinal tract for the leg muscles (prolongation of the CMCT and CCCT). Together these findings indicate the involvement of the sensorimotor tracts in both the CNS and peripheral nervous system, mainly those for the lower extremities.

Muscular atrophy in McLeod syndrome has been explained by motor axonal neuropathy. A muscle biopsy can detect a combination of neurogenic and myogenic changes, with the former being more obvious [3]. Sensory symptoms have also been attributed to sensory axonal neuropathy [11, 13]. However, the pathogenesis of the peripheral neuropathy remains to be determined.

This is the first demonstration of the involvement of the central sensorimotor tracts in McLeod syndrome. The findings of this study suggest the presence of length-dependent axonal degeneration of the sensorimotor tract fibers in McLeod syndrome. This is especially interesting given that a previous pathological study found that model knockout mice of McLeod syndrome showed axonopathy in the spinal cord and the sciatic nerve [4]. The present findings provide clinical proof for these experimental results, which is important for understanding the distribution of this multisystem disorder.

A unique finding of the present case study is that the TMS technique revealed the involvement of the corticospinal tract even in a patient whose plantar responses were indifferent. When both the corticospinal tract and peripheral nerves are damaged, Babinski signs may not be observed, because the peripheral pathology masks the central pathology. TMS can allow evaluation of corticospinal tract function even in this situation [9]. The lack of evidence for corticospinal tract involvement in the previous clinical reports might be due to coexisting peripheral neuropathy, which is common in McLeod syndrome. Similarly, measuring SEPs is also useful for detecting the involvement of central sensory pathways in patients with peripheral neuropathy.

In conclusion, the results obtained in this study suggest that McLeod syndrome involves the central sensorimotor tracts in addition to peripheral nerves. This involvement of central sensorimotor tracts for the legs might be masked by the presence of peripheral neuropathy in this disorder.

## Data Availability

Not applicable.

## Abbreviations

CCCT: Cortico-conus motor conduction time
CECT: Cauda equina conduction time
CMCT: Central motor conduction time
CNS: Central nervous system
CSCT: Central sensory conduction time
FDI: First-dorsal interosseous
MEP: Motor-evoked potential
MRI: Magnetic resonance image
MUP: Motor unit potential
NCS: Nerve conduction study
SEP: Somatosensory-evoked potential
TA: Tibialis anterior
TMS: Transcranial magnetic stimulation
